# Supplementary material for: Unveiling the Bioactive Potential of the Invasive Jellyfish Phyllorhiza punctata Through Integrative Transcriptomic and Proteomic Analyses
Source: Biomolecules. 2025 Aug 4;15(8):1121. doi: 10.3390/biom15081121 (PMC12383608; doi:10.3390/biom15081121)
Supplement: Supplementary file 1 [file biomolecules-15-01121-s001.zip › Suplementary Material/FileS3/PEPFOLD4/XP_065052063_14066/clusters.html]

 PEP-FOLD clusters


Download archive of all models  here   

|  |  |  |
| --- | --- | --- |
| # | file | sOPEP |
| # cluster 1 - Size: 3 |  |  |
|  | XP\_065052063\_14066-model1 | -7.37223 |
|  | XP\_065052063\_14066-model1.1 | -6.15634 |
|  | XP\_065052063\_14066-model1.2 | -5.71721 |
| # cluster 2 - Size: 1 |  |  |
|  | XP\_065052063\_14066-model2 | -7.14548 |
| # cluster 3 - Size: 4 |  |  |
|  | XP\_065052063\_14066-model3 | -6.38071 |
|  | XP\_065052063\_14066-model3.1 | -6.1369 |
|  | XP\_065052063\_14066-model3.2 | -5.83338 |
|  | XP\_065052063\_14066-model3.3 | -5.63111 |
| # cluster 4 - Size: 5 |  |  |
|  | XP\_065052063\_14066-model4 | -6.37519 |
|  | XP\_065052063\_14066-model4.1 | -5.94933 |
|  | XP\_065052063\_14066-model4.2 | -5.85704 |
|  | XP\_065052063\_14066-model4.3 | -5.62076 |
|  | XP\_065052063\_14066-model4.4 | -5.50162 |
| # cluster 5 - Size: 2 |  |  |
|  | XP\_065052063\_14066-model5 | -6.2858 |
|  | XP\_065052063\_14066-model5.1 | -5.57973 |
| # cluster 6 - Size: 2 |  |  |
|  | XP\_065052063\_14066-model6 | -6.19742 |
|  | XP\_065052063\_14066-model6.1 | -5.55154 |
| # cluster 7 - Size: 11 |  |  |
|  | XP\_065052063\_14066-model7 | -6.16094 |
|  | XP\_065052063\_14066-model7.1 | -6.11497 |
|  | XP\_065052063\_14066-model7.2 | -5.92239 |
|  | XP\_065052063\_14066-model7.3 | -5.86751 |
|  | XP\_065052063\_14066-model7.4 | -5.81124 |
|  | XP\_065052063\_14066-model7.5 | -5.77297 |
|  | XP\_065052063\_14066-model7.6 | -5.74891 |
|  | XP\_065052063\_14066-model7.7 | -5.68544 |
|  | XP\_065052063\_14066-model7.8 | -5.67376 |
|  | XP\_065052063\_14066-model7.9 | -5.64521 |
|  | XP\_065052063\_14066-model7.10 | -5.50863 |
| # cluster 8 - Size: 2 |  |  |
|  | XP\_065052063\_14066-model8 | -6.14115 |
|  | XP\_065052063\_14066-model8.1 | -5.57934 |
| # cluster 9 - Size: 1 |  |  |
|  | XP\_065052063\_14066-model9 | -6.05061 |
| # cluster 10 - Size: 13 |  |  |
|  | XP\_065052063\_14066-model10 | -6.04879 |
|  | XP\_065052063\_14066-model10.1 | -6.03231 |
|  | XP\_065052063\_14066-model10.2 | -6.01678 |
|  | XP\_065052063\_14066-model10.3 | -6.01124 |
|  | XP\_065052063\_14066-model10.4 | -5.98093 |
|  | XP\_065052063\_14066-model10.5 | -5.97288 |
|  | XP\_065052063\_14066-model10.6 | -5.94594 |
|  | XP\_065052063\_14066-model10.7 | -5.93932 |
|  | XP\_065052063\_14066-model10.8 | -5.85237 |
|  | XP\_065052063\_14066-model10.9 | -5.76677 |
|  | XP\_065052063\_14066-model10.10 | -5.664 |
|  | XP\_065052063\_14066-model10.11 | -5.6294 |
|  | XP\_065052063\_14066-model10.12 | -5.62856 |
| # cluster 11 - Size: 3 |  |  |
|  | XP\_065052063\_14066-model11 | -6.04468 |
|  | XP\_065052063\_14066-model11.1 | -5.60012 |
|  | XP\_065052063\_14066-model11.2 | -5.50434 |
| # cluster 12 - Size: 2 |  |  |
|  | XP\_065052063\_14066-model12 | -5.9244 |
|  | XP\_065052063\_14066-model12.1 | -5.72992 |
| # cluster 13 - Size: 1 |  |  |
|  | XP\_065052063\_14066-model13 | -5.87546 |
| # cluster 14 - Size: 1 |  |  |
|  | XP\_065052063\_14066-model14 | -5.86702 |
| # cluster 15 - Size: 1 |  |  |
|  | XP\_065052063\_14066-model15 | -5.86407 |
| # cluster 16 - Size: 11 |  |  |
|  | XP\_065052063\_14066-model16 | -5.85306 |
|  | XP\_065052063\_14066-model16.1 | -5.83844 |
|  | XP\_065052063\_14066-model16.2 | -5.76663 |
|  | XP\_065052063\_14066-model16.3 | -5.71888 |
|  | XP\_065052063\_14066-model16.4 | -5.69804 |
|  | XP\_065052063\_14066-model16.5 | -5.69498 |
|  | XP\_065052063\_14066-model16.6 | -5.68429 |
|  | XP\_065052063\_14066-model16.7 | -5.62499 |
|  | XP\_065052063\_14066-model16.8 | -5.61285 |
|  | XP\_065052063\_14066-model16.9 | -5.56767 |
|  | XP\_065052063\_14066-model16.10 | -5.40655 |
| # cluster 17 - Size: 5 |  |  |
|  | XP\_065052063\_14066-model17 | -5.84853 |
|  | XP\_065052063\_14066-model17.1 | -5.74377 |
|  | XP\_065052063\_14066-model17.2 | -5.6404 |
|  | XP\_065052063\_14066-model17.3 | -5.61329 |
|  | XP\_065052063\_14066-model17.4 | -5.54761 |
| # cluster 18 - Size: 2 |  |  |
|  | XP\_065052063\_14066-model18 | -5.84538 |
|  | XP\_065052063\_14066-model18.1 | -5.69771 |
| # cluster 19 - Size: 6 |  |  |
|  | XP\_065052063\_14066-model19 | -5.8453 |
|  | XP\_065052063\_14066-model19.1 | -5.74452 |
|  | XP\_065052063\_14066-model19.2 | -5.64 |
|  | XP\_065052063\_14066-model19.3 | -5.5041 |
|  | XP\_065052063\_14066-model19.4 | -5.4849 |
|  | XP\_065052063\_14066-model19.5 | -5.40224 |
| # cluster 20 - Size: 1 |  |  |
|  | XP\_065052063\_14066-model20 | -5.83817 |
| # cluster 21 - Size: 4 |  |  |
|  | XP\_065052063\_14066-model21 | -5.82759 |
|  | XP\_065052063\_14066-model21.1 | -5.77833 |
|  | XP\_065052063\_14066-model21.2 | -5.68933 |
|  | XP\_065052063\_14066-model21.3 | -5.58353 |
| # cluster 22 - Size: 2 |  |  |
|  | XP\_065052063\_14066-model22 | -5.80984 |
|  | XP\_065052063\_14066-model22.1 | -5.52815 |
| # cluster 23 - Size: 1 |  |  |
|  | XP\_065052063\_14066-model23 | -5.79123 |
| # cluster 24 - Size: 2 |  |  |
|  | XP\_065052063\_14066-model24 | -5.71929 |
|  | XP\_065052063\_14066-model24.1 | -5.67387 |
| # cluster 25 - Size: 1 |  |  |
|  | XP\_065052063\_14066-model25 | -5.71584 |
| # cluster 26 - Size: 1 |  |  |
|  | XP\_065052063\_14066-model26 | -5.70085 |
| # cluster 27 - Size: 1 |  |  |
|  | XP\_065052063\_14066-model27 | -5.68458 |
| # cluster 28 - Size: 1 |  |  |
|  | XP\_065052063\_14066-model28 | -5.64385 |
| # cluster 29 - Size: 2 |  |  |
|  | XP\_065052063\_14066-model29 | -5.63736 |
|  | XP\_065052063\_14066-model29.1 | -5.63154 |
| # cluster 30 - Size: 2 |  |  |
|  | XP\_065052063\_14066-model30 | -5.61694 |
|  | XP\_065052063\_14066-model30.1 | -5.53192 |
| # cluster 31 - Size: 2 |  |  |
|  | XP\_065052063\_14066-model31 | -5.609 |
|  | XP\_065052063\_14066-model31.1 | -5.47659 |
| # cluster 32 - Size: 1 |  |  |
|  | XP\_065052063\_14066-model32 | -5.586 |
| # cluster 33 - Size: 1 |  |  |
|  | XP\_065052063\_14066-model33 | -5.51 |
| # cluster 34 - Size: 1 |  |  |
|  | XP\_065052063\_14066-model34 | -5.50644 |
| # cluster 35 - Size: 1 |  |  |
|  | XP\_065052063\_14066-model35 | -5.46395 |
|  |  |  |
